# A Screen-Printed Sensor Coupled with Flow System for Quantitative Determination of a Novel Promising Anticancer Agent Candidate

**DOI:** 10.3390/s20185217

**Published:** 2020-09-13

**Authors:** Katarzyna Tyszczuk-Rotko, Jędrzej Kozak, Małgorzata Sztanke, Krzysztof Sztanke, Ilona Sadok

**Affiliations:** 1 Faculty of Chemistry, Institute of Chemical Sciences, Maria Curie-Skłodowska University in Lublin, 20-031 Lublin, Poland; jedrekkozak@onet.pl; 2 Chair and Department of Medical Chemistry, Medical University of Lublin, 20-093 Lublin, Poland; malgorzata.sztanke@umlub.pl; 3 Laboratory of Bioorganic Synthesis and Analysis, Chair and Department of Medical Chemistry, Medical University of Lublin, 20-093 Lublin, Poland; krzysztofsztanke@umlub.pl; 4 Laboratory of Separation and Spectroscopic Method Application, Centre for Interdisciplinary Research, Faculty of Science and Health, The John Paul II Catholic University of Lublin, 20-708 Lublin, Poland; ilona.sadok@kul.pl

**Keywords:** anticancer agent candidate, voltammetric analysis, screen-printed sensor, flow system, liquid chromatography

## Abstract

A carbon nanofibers modified screen-printed carbon sensor (SPCE/CNFs) was applied for the determination of a novel promising anticancer agent candidate (ethyl 8-(4-methoxyphenyl)-4-oxo-4,6,7,8-tetrahydroimidazo[2,1-*c*][1,2,4]triazine-3-carboxylate, EIMTC) using square-wave voltammetry (SWV). It is the first method for the quantitative determination of EIMTC. The modified screen-printed sensor exhibited excellent electrochemical activity in reducing EIMTC. The peak current of EIMTC was found to be linear in two concentration ranges of 2.0 × 10^−9^ – 2.0 × 10^−8^ mol L^−1^ and 2.0 × 10^−8^ – 2.0 × 10^−7^ mol L^−1^, with a detection limit of 5.0 × 10^−10^ mol L^−1^. The connection of flow-cell for the SPCE/CNFs with SWV detection allowed for the successful determination of EIMTC in human serum samples. Ultra-high-performance liquid chromatography coupled to electrospray ionization triple quadrupole mass spectrometry (UHPLC-ESI-MS/MS) acted as a comparative method in the serum samples analysis.

## 1. Introduction

**Figure 1:**
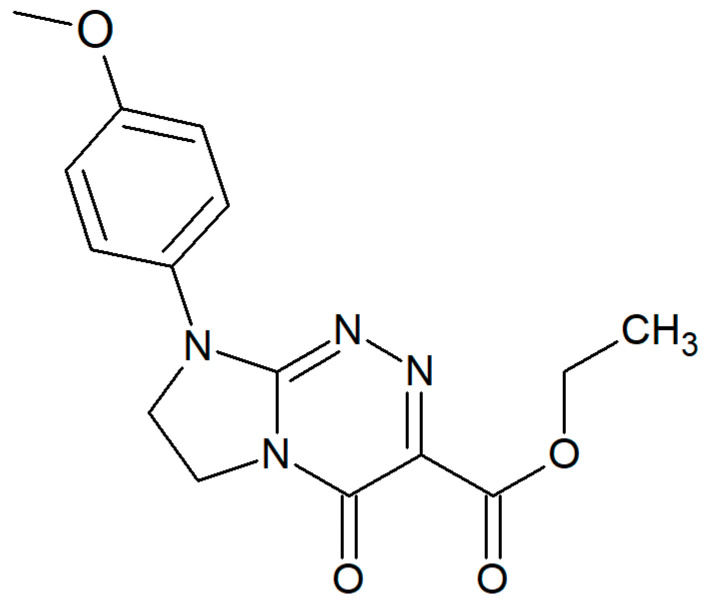
The structure of the investigated EIMTC.

Ethyl 8-(4-methoxyphenyl)-4-oxo-4,6,7,8-tetrahydroimidazo[2,1-*c*][1,2,4]triazine-3-carboxylate (PubChem CID: 11507951), namely EIMTC (Figure 1), is an interesting electroactive small molecule for our current electrochemical research needs. EIMTC has been shown to possess a fully defined molecular structure, and a low toxicity in vitro and in vivo [1,2,3]. The same compound has been reported as the most promising innovative nucleobase-like structure (belonging to a class of fused azaisocytosine-containing congeners) with a broad spectrum of anticancer activity (i.e., in multiple myeloma cells and tumor cells of the cervix and breast) as well as good bioavailability and permeability properties [3]. Its methods of synthesis, a complete structural, physico-chemical and pharmacological characterization, have been published earlier together with all the derivatives belonging to the same class of molecules [1,2,3]. EIMTC has been proposed as a novel anticancer drug candidate with potential applicability in the treatment of multiple myelomas due to its remarkable antiproliferative activity in human multiple myeloma cells (i.e., resistant as well as susceptible to thalidomide) combined with a less toxic effect on normal cells. In addition, this potential anticancer drug candidate has been shown to induce growth arrest in cancer cells and evoke higher necrosis rates in tumor than in non-tumoral cells of the same epithelial origin [3]. 

Despite the above-mentioned utilities, no analytical method of EIMTC determination has been developed as of yet. Therefore, the present paper is aimed at developing and optimizing the first electrochemical procedure that allows the quantitative determination of this novel anticancer agent candidate. In the studies, unmodified and modified with carbon nanofibers screen-printed carbon sensors (SPCE and SPCE/CNFs, respectively) were applied for the voltammetric determination of EIMTC.

It is worth mentioning that electrochemical methods, including among others voltammetry, offer a high sensitivity with relatively low-cost instrumentation and analysis [4]. In turn, due to the mass production nature, availability and low cost of screen-printed electrodes, they are now a good approach to the preparation of voltammetric sensors [5,6,7,8,9]. Moreover, the application of nanomaterials together with screen-printing fabrication is a very actual subject of research of particular importance especially for environmental monitoring and medical diagnostics, but also in other fields of analytical applications [10]. One of the attractive carbon nanomaterials for the sensor fabrication are carbon nanofibers (CNFs). The beginning of their electrochemical applications can be dated to early 2000s [11]. Until now, CNFs have found numerous applications, which are connected with their attractive properties, especially their large number of edge-plane sites and their high surface active group-to-volume ratio [12,13]. Furthermore, CNFs can be easily functionalized to suit a particular detection mechanism [13].

## 2. Materials and Methods

### 2.1. Instrumentations

All voltammetric measurements were carried out using an electrochemical potentiostat (µAutolab, Eco Chemie, Utrecht, The Netherlands) connected to a personal computer operated by GPES 4.9 software. The 4 mm diameter sensors (SPCE – ref. 110 and SPCE/CNFs – ref. 110CNF) were provided by DropSens, Llanera, Spain. The sensors were immersed in a classic quartz cell (volume 10 mL) or a commercially available methacrylate wall-jet flow-cell (ref. FLWCL, DropSens, Llanera, Spain). The experiments on flow system were carried out using a peristaltic pump type MS-CA (Ismatec, Wertheim, Germany) and sample injection with a 500 μL sample loop (Valco Instruments Co. Inc., Houston, USA). 

The images of sensors surface were recorded using a high-resolution scanning electron microscope Quanta 3D FEG (FEI, Hillsboro, USA). 

The UHPLC-ESI-MS/MS system was used consisting of a model 1290 infinity ultra-high performance liquid chromatograph (Agilent Technologies, USA) connected to a 6460 triple quadrupole mass spectrometer (Agilent Technologies, USA) equipped with an electrospray ion source (Agilent Jet Stream) operating in the positive ion mode. The instrument was controlled using Agilent MassHunter Acquisition software v.B.08. The data were analyzed by Agilent MassHunter Quantitative Analysis software v.B.07. The chromatography was performed on a Zorbax Eclipse Plus-C18 Rapid resolution HT column (2.1 mm × 50 mm × 1.8 µm) protected by a Zorbax Eclipse Plus-C18 Narrow Bore Guard Column (2.1 mm × 12.5 mm × 5 µm), both purchased from Agilent Technologies. 

### 2.2. Chemicals 

The EIMTC was synthesized from 2-hydrazinylidene-1-(4-methoxyphenyl)imidazolidine and diethyl 2-hydroxyiminomalonate according to the synthetic approach published earlier [1,2]. The chemical structure of the analyzed compound was determined by consistent spectroscopic data (including IR, ^1^H NMR, EI-MS), whereas its high level of purity was confirmed by a sharp melting point (150–151 °C) and found elemental analyses being within ±0.4% of the theoretical values for each element analyzed. 

The solutions of sulfuric acid, acetic acid, and acetate buffers of different pH were prepared from Sigma-Aldrich reagents. The Merck (Darmstadt, Germany) standard solutions of Ca^2+^, Mg^2+^, Fe^3+^, Cl^−^ as well as Sigma-Aldrich (Saint Louis, USA) reagents (adenine, dopamine, epinephrine, glucose, uric acid, ascorbic acid and estradiol) were used in interference studies. For voltammetric measurements, 1.0 × 10^−3^ mol L^−1^ solution of EIMTC was prepared in *N,N*-dimethylformamide (Sigma-Aldrich, Saint Louis, USA). For UHPLC-ESI-MS/MS analysis, methanol (hypergrade, Merck, Darmstadt, Germany) and formic acid (LC-MS, Sigma-Aldrich, Saint Louis, USA) were used.

### 2.3. Sample Preparation

The 100 µL of human serum sample 100-times diluted in ultrapure water (Sigma-Aldrich, Saint Louis, USA) spiked with an appropriate concentration of EIMTC was transferred to a centrifugal tube, mixed with 25 µL of 7.5% (*w*/*v*) trichloroacetic acid solution (TCA, Sigma-Aldrich, Saint Louis, USA) for protein precipitation, vortexed well, and centrifuged at 14,000× *g* for 15 min at 4 °C (5415R Centrifuge, Eppendorf, Germany). The collected supernatant was centrifuged once again (14,000× *g*, 15 min, 4 °C), and the clear aliquot was analyzed in triplicate by the SWV and UHPLC-ESI-MS/MS methods.

### 2.4. SWV Analysis 

In the case of measurements performed in a classic electrochemical cell, the standard solution of EIMTC was added to the supporting electrolyte of 0.075 mol L^−1^ H_2_SO_4_, mixed for 45 s (open circuit potential), and SWV curves were registered. In flow system, in the first step of analysis 2 mol L^−1^ H_2_SO_4_ for 55 s was directed through the cell in order to clean the electrode surface. Then, 500 μL of 0.075 mol L^−1^ solution of H_2_SO_4_ containing an appropriate concentration of EIMTC or the sample added to 0.075 mol L^−1^ solution of H_2_SO_4_ was injected. After 55 s (open circuit potential) from the moment of sample injection, SWV measurements were carried out. The flow rates of each solution were about 3 mL min^−1^. For SWV, the optimum parameters are as follows: initial potential of −0.2 V, final potential of −0.9 V, frequency (*f*) of 50 Hz, square-wave amplitude (Δ*E_A_*) of 50 mV, and step potential (Δ*E_step_*) of 7 mV. The signal of EIMTC was measured after subtracting the background.

### 2.5. UHPLC-ESI-MS/MS Analysis 

The ionization parameters were as follows: nebulizer: 35 psi; gas temperature: 300 °C; gas flow: 10 L min^−1^; sheath gas temperature: 325 °C; sheath gas flow: 10 L min^−1^; and capillary voltage: 4000 V. The mobile phase consisted of two solutions: A (0.1% (*v*/*v*) formic acid in water) and B (methanol). The gradient program was as follows: 0–5 min—5% to 70% B; 5–7 min—5% B (column re-equilibration). The column temperature and mobile phase rate were 40°C and 0.3 mL min^−1^, respectively. The injection volume was 5 µL. The analyte’s ions were monitored in Multiple Reaction Monitoring (MRM) mode. MRM transitions: *m*/*z* 317 > 289 (quantifier, fragmentor: 140 V; collision energy: 20 eV) and *m*/*z* 317 > 245 (qualifier, fragmentor: 140 V; collision energy: 22 eV).

## 3. Results and Discussion

### 3.1. Voltammetric Behaviour of EIMTC

**Figure 2:**
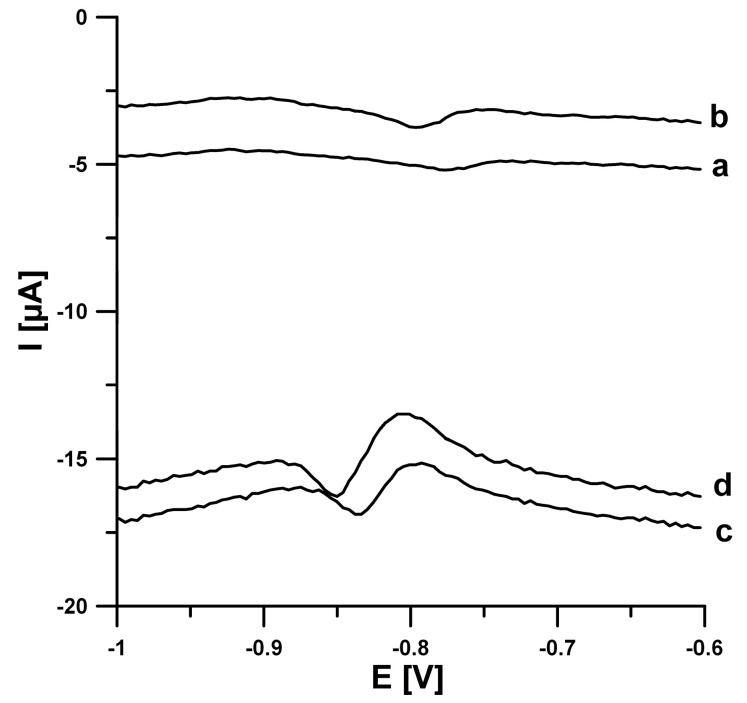
Square-wave voltammograms of 5.0 × 10^−7^ mol L^−1^ (a and c) and 1.0 × 10^−6^ mol L^−1^ (b and d) EIMTC in 0.1 mol L^−1^ acetate buffer solution of pH 4.5 at the screen-printed carbon sensor (SPCE) (a and b) and carbon nanofibers modified screen-printed carbon sensors (SPCE/CNFs) (c and d). The SWV parameters: open circuit potential, *t* of 45 s, initial *E* of −0.6 V, final *E* of −1.0 V, *f* of 50 Hz, Δ*E_A_* of 25 mV, and Δ*E_step_* of 4 mV.

**Figure 3:**
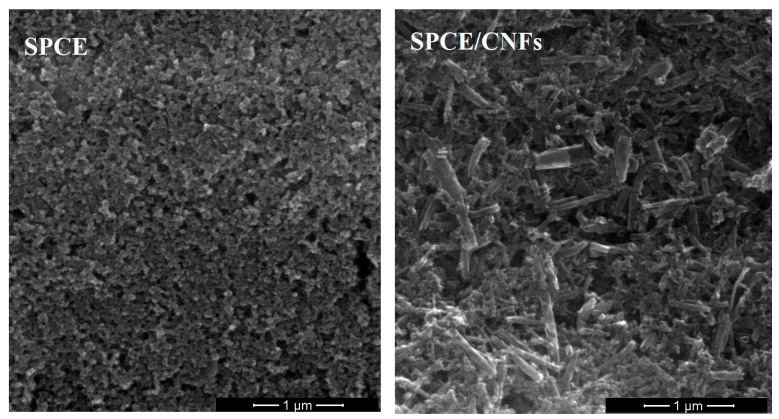
SEM images of SPCE and SPCE/CNFs.

The electrochemical behavior of EIMTC at the unmodified SPCE and carbon nanofibers modified SPCE is shown in Figure 2. Before SWV curve registration, the solution was mixed for 45 s (open circuit potential). It can be seen that the reduction peaks of EIMTC at the SPCE were weak, while the EIMTC responses were considerably improved at the SPCE/CNFs. The reason for better performance of SPCE/CNFs was explained in our earlier works [14,15]. In those papers, the active surface areas of the SPCE and SPCE/CNFs were calculated using cyclic voltammetric studies in 0.1 mol L^−1^ solution of KCl and 5.0 × 10^−3^ mol L^−1^ K_3_[Fe(CN)_6_] and the Randles–Sevcik equation. It was found that modification of the electrode increases its active surface (Figure 3). 

**Figure 4:**
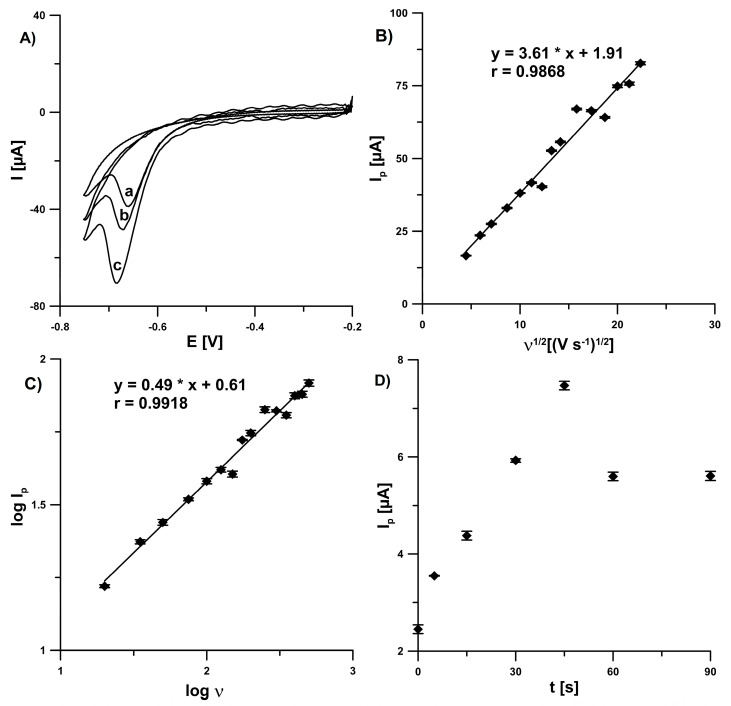
(**A**) Cyclic voltammograms of 1.0 × 10^−5^ mol L^−1^ EIMTC at ν of 50 (a), 100 (b) and 150 mV s^−1^ (c). The dependence between (**B**) *Ip* and *v*^1/2^ and (**C**) log*Ip* and log*v*. (**D**) Effect of *t* on *I_p_* of 2.0 × 10^−7^ mol L^−1^ EIMTC (open circuit potential). The average values of *I_p_* are shown with the standard deviation of n = 3.

In the next part of the experiments, cyclic voltammograms (CVs) were recorded in the range of −0.2–(−0.75)V to get information about the behavior of EIMTC at the SPCE/CNFs. Figure 4A represents CV curves recorded for 1.0 × 10^−5^ mol L^−1^ EIMTC in 0.1 mol L^−1^ acetate buffer of pH 4.5 with scan rates (*ν*) of 50, 100, and 150 mV s^−1^. As it can be seen, well-defined irreversible reduction peaks of EIMTC were obtained. To ascertain the effect of scan rate on the reduction peak current of EIMTC, the scan rate studies were carried out in the range of 20–500 mV s^−1^. The linearity of peak current (*I_p_*) of EIMTC vs. *ν*^1/2^ plot (Figure 4B) indicated that the reduction of EIMTC at the SPCE/CNFs is diffusion controlled, which was further confirmed by the slope value (0.49) of log*I_p_* vs. log*ν* plot (Figure 4C) [16]. Furthermore, the *I_p_* values of 2.0 × 10^−7^ mol L^−1^ EIMTC were almost stable during changes in the potential (0–(−0.8)V) applied to the electrode, which also confirmed that the reduction of EIMTC is diffusion-controlled at the SPCE/CNFs. While the potential does not significantly affect the EIMTC signal, mixing of the solution prior the SWV curves registration (open circuit potential) is very important. As can be seen in Figure 4D, the *I_p_* value of 2.0 × 10^−7^ mol L^−1^ EIMTC reached maximum at the solution mixing time (*t*) of 45 s. It is connected with the facilitated diffusion of EIMTC molecules to the electrode surface during solution mixing [17]. 

### 3.2. Effect of pH 

**Figure 5:**
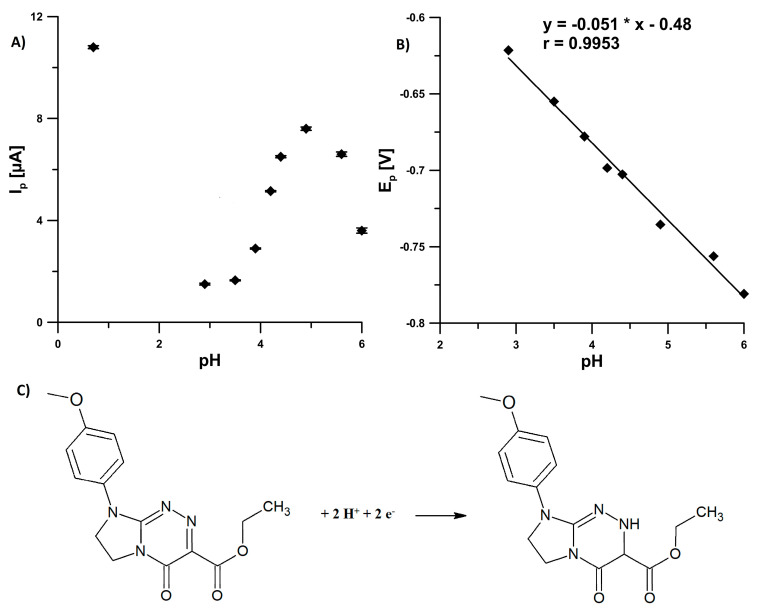
Effect of pH on: (**A**) *I_p_* and (**B**) *E_p_* of 5.0 × 10^−7^ mol L^−1^ EIMTC (open circuit potential, *t* of 45 s). The SWV parameters: initial *E* of −0.2 V, final *E* of −0.9 V, *f* of 50 Hz, Δ*E_A_* of 25 mV and Δ*E_step_* of 4 mV. (**C**) The mechanism proposed for the reduction of EIMTC. The average values of *I_p_* are shown with the standard deviation of n = 3.

The pH dependence of 5.0 × 10^−7^ mol L^−1^ EIMTC at the SPCE/CNFs was investigated using 0.1 mol L^−1^ sulfuric acid, acetic acid, and acetate buffer (pH of 3.5–6.0). From the plot of *I_p_* of EIMTC vs. pH (Figure 5A) it is clear that peak current is affected by the pH value. The best result with respect to sensitivity accompanied with a well-defined response was obtained in the H_2_SO_4_ solution, so this solution was used in further studies. The linearity of peak potential (*E_p_*) of EIMTC vs. pH plot (Figure 5B) was obtained in the pH range of 2.9–6.0 (*r* = 0.9950). The slope of the equation was found to be 51 mV pH^−1^. This closeness of the slope to the expected theoretical value of 59 mV pH^−1^ suggested that the number of electrons is equal to the hydrogen ions taking part in the electrode reaction. 

EIMTC is a small molecule possessing in a privileged triazinone scaffold the azomethine grouping of ketimine type (C=N) that can be reduced electrochemically. The reduction of this C=N grouping in the analyzed molecule, leads to the protonated CH–NH grouping. This is consistent with previous studies on electrochemical behavior of monocyclic as well as fused triazinones [18,19]. The one-step reduction process at the SPCE/CNFs surface occurs through an electron-gain mechanism, including the transfer of two electrons and two protons (Figure 5C). 

Moreover, the effect of selected supporting electrolyte (H_2_SO_4_) concentration (0.025–0.2 mol L^−1^) on the peak current of 5.0 × 10^−7^ mol L^−1^ EIMTC at the SPCE/CNFs was studied. The highest signals were obtained at a concentration of 0.075 mol L^−1^ and at higher concentrations of H_2_SO_4_ the EIMTC signals are almost stable. Therefore, 0.075 mol L^−1^ H_2_SO_4_ solution was selected for further experiments. 

### 3.3. Effect of SWV Parameters

**Figure 6:**
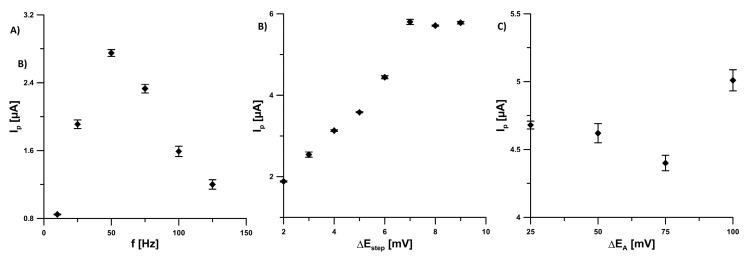
Effect of (**A**) *f* (10–125 Hz), (**B**) Δ*E_step_* (2–9 mV) and (**C**) Δ*E_A_* (25–100 mV) on 5.0 × 10^−7^ mol L^−1^ EIMTC responses. The average values of *I_p_* are shown with the standard deviation of n = 3.

The dependence of the reduction in peak current (*I_p_*) of 5.0 × 10^−7^ mol L^−1^ EIMTC on the square-wave frequency (*f*) was studied in the range of 10–125 Hz at the SPCE/CNFs (Figure 6A). The *I_p_* values were found to increase linearly, with increasing *f* to 50 Hz, and then to decrease. That is why *f* to 50 Hz was chosen for further studies. Next, the effect of step potential (Δ*E_step_*) on the 5.0 × 10^−7^ mol L^−1^ EIMTC signals was examined from 2 to 9 mV (*f* to 50 Hz and Δ*E_A_* of 25 mV). The highest EIMTC signal was obtained at Δ*E_step_* of 7 mV (Figure 6B). Furthermore, the influence of square-wave amplitude (*ΔE_A_*) on the 5.0 × 10^−7^ mol L^−1^ EIMTC responses was examined in the rage of 25–100 mV (Figure 6C, *f* to 50 Hz and Δ*E_step_* of 7 mV). The maximum value of EIMTC signal (5.0 µA) was achieved at *ΔE_A_* of 100 mV. However, due to the much lower background current and a slight difference in EIMTC peak current (4.6 vs. 5.0 µA), *ΔE_A_* of 50 mV was selected for further experiments. 

### 3.4. Interferences 

The presence of common ions (Ca^2+^, Mg^2+^, Fe^3+^, and Cl^−^) and organic compounds (uric acid, ascorbic acid, glucose, adenine, dopamine, epinephrine, and estradiol) in human serum samples may alter electrochemical EIMTC signals and consequently affect the selectivity. The tolerance concentration ratios with respect to 5.0 × 10^−7^ mol L^−1^ EIMTC for interferences at 10% level were examined. The results given in Table 1 show that Fe^3+^, estradiol, and ascorbic acid have a maximum effect on the determination of EIMTC. 

The preliminary analysis of human serum samples spiked with 5.0 × 10^−8^ mol L^−1^ EIMTC in the classical electrochemical cell did not bring expected results. No EIMTC signals were observed. This signal attenuation was related to the serum sample matrix. Therefore, flow-injection analysis in a commercially available wall-jet flow-cell was proposed to resolve this problem. It is well known that the electrochemical detection under hydrodynamically controlled conditions reveals some benefits. Among others, the shear forces of the flowing liquid continuously regenerate the working electrode surface and remove reaction products [20]. Consequently, the selectivity under flow analytical conditions can be improved, which was confirmed by our research (see Section 3.6). 

### 3.5. Calibration Curve in Flow System, Precision and Reproducibility

**Figure 7:**
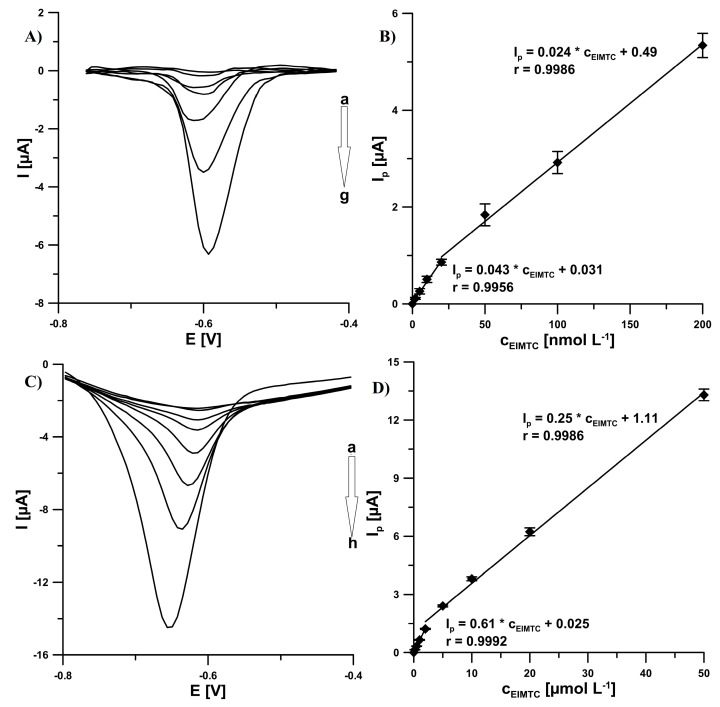
(**A**) SWV curves registered in flow system at the SPCE/CNFs in 0.075 mol L^−1^ H_2_SO_4_ solution containing increasing concentrations of EIMTC: 2.0 × 10^−9^ (a), 5.0 × 10^−9^ (b), 1.0 × 10^−8^ (c), 2.0 × 10^−8^ (d), 5.0 × 10^−8^ (e), 1.0 × 10^−7^ (f), 2.0 × 10^−7^ (g) mol L^−1^. (**B**) Linear ranges of the calibration graph of EIMTC in flow system at the SPCE/CNFs. (**C**) SWV curves registered in flow system at the SPCE in 0.075 mol L^−1^ H_2_SO_4_ solution containing increasing concentrations of EIMTC: 2.0 × 10^−7^ (a), 5.0 × 10^−7^ (b), 1.0 × 10^−6^ (c), 2.0 × 10^−6^ (d), 5.0 × 10^−6^ (e), 1.0 × 10^−5^ (f), 2.0 × 10^−5^ (g), 5.0 × 10^−5^ (h) mol L^−1^. (**D**) Linear ranges of the calibration graph of EIMTC in flow system at the SPCE. The average values of *I_p_* are shown with the standard deviation of n = 9. After 55 s (open circuit potential) from the moment of sample injection (500 µL sample loop), SWV measurements were carried out. The SWV parameters: initial *E* of −0.2 V, final *E* of −0.9 V, *f* of 50 Hz, Δ*E_A_* of 50 mV, and Δ*E_step_* of 7 mV.

The SPCE/CNFs sensor coupled with flow system was used for the quantitative determination of a novel promising anticancer agent candidate EIMTC. The flow conditions and SWV parameters are described in Section 2.4. The responses were linear with EIMTC concentrations ranging from 2.0 × 10^−9^ to 2.0 × 10^−8^ mol L^−1^ and 2.0 × 10^−8^ to 2.0 × 10^−7^ mol L^−1^ (Figure 7A,B). The limits of detection (LOD) and quantification (LOQ) were determined 5.0 × 10^−10^ and 1.7 × 10^−9^ mol L^−1^, respectively, according to the definitions of LOD = 3SD_a_/b and LOQ = 10SD_a_/b (SD_a_—standard deviation of intercept (n = 3); b—slope of calibration curve) [21].

The relative standard deviation (RSD) values of 2.9% and 5.2% calculated for ten repeated measurements of 2.0 × 10^−8^ and 2.0 × 10^−7^ mol L^−1^ EIMTC at the SPCE/CNFs, respectively, confirmed the satisfactory precision of the signals at the SPCE/CNFs. In turn, the RSD values of 3.4% and 4.7% (n = 9) calculated for the same EIMTC concentrations but using three electrodes, indicated the acceptable reproducibility of the sensor.

Moreover, in order to confirm the advantage of using the SPCE/CNFs instead of the unmodified SPCE in the EIMTC determinations, the voltammetric measurements to the calibration curve were also made at the SPCE (Figs. 7C and 7D). The calibration curve was linear in two ranges from 2.0 × 10^−7^ to 2.0 × 10^−6^ mol L^−1^ and from 2.0 × 10^−6^ to 5.0 × 10^−5^ mol L^−1^, with detection and quantification limits of 3.4 × 10^−8^ and 1.1 × 10^−7^ mol L^−1^, respectively. The comparison demonstrates that the SPCE/CNFs provides two orders of magnitude lower detection and quantifications limits.

### 3.6. Serum Samples Assay in Flow System

To establish the utility of the developed flow voltammetric procedure in biological samples, EIMTC was determined in the spiked human serum samples. The calibration graph was used for the determination of spiked serum samples. The results obtained for the SWV and UHPLC-ESI-MS/MS analysis are listed in Table 2. The recovery obtained was 97.2% and 99.0%. The relative error values of 3.0% and 7.2% show satisfactory agreement with the cooperative UHPLC-ESI-MS/MS method. It was also confirmed by the t-Student test. The calculated *t* values (*t_exp._*) are 1.98 and 0.71, which is below the critical value equal to 2.78 (level of significance of 0.05, number of degrees of freedom (*f*) of 4, *f* = *n_1_*+ *n_2_* – 2) [22]. Moreover, the results indicate that there is no significant effect of the sample serum matrix on the voltammetric EIMTC signal, and the developed SWV procedure in a flow system is feasible for EIMTC analysis in real biological samples.

## 4. Conclusions

In the present studies, the first analytical method was proposed for sensitive and selective determination of a novel promising anticancer agent candidate (EIMTC) using the carbon nanofibers modified screen-printed carbon sensor. The modified sensor showed great improvement to the EIMTC reduction electrode process compared to the unmodified sensor (LOD: 5.0 × 10^−10^ mol L^−1^ vs. 3.4 × 10^−8^ mol L^−1^ and 1.7 × 10^−9^ mol L^−1^ vs. 1.1 × 10^−7^ mol L^−1^, respectively). The reason for enhanced detection performance at the SPCE/CNFs is related to an increase in the number of active sites, as we showed earlier [14,15]. Furthermore, the electrochemical responses of EIMTC at the SPCE/CNFs were characterized by the CV technique and the results indicated that the reduction process of EIMTC is diffusion-controlled. The diffusion of EIMTC molecules to the electrode surface was facilitated during solution mixing before the SWV curve registration. Moreover, the flow system successfully resolved the problem with the influence of the human serum matrix on the EIMTC signal. The application of the developed voltammetric procedure for analysis of human serum samples was successfully demonstrated. The results show satisfactory agreement with the cooperative UHPLC-ESI-MS/MS method. The proposed method is characterized by a wide linear range, low detection and quantification limits, as well as satisfactory precision and reproducibility.

## Figures and Tables

**Table 1:** Results of interference studies for the determination of 5.0 × 10^−7^ mol L^−1^ EIMTC at the SPCE/CNFs.

Species	Tolerance Limits
Ca^2+^	50
Mg^2+^, Cl^−^, uric acid, glucose, adenine, dopamine	5
epinephrine	2.5
Fe^3+^, estradiol, ascorbic acid	0.5

**Table 2:** Results obtained for EIMTC in human serum samples using SWV at the SPCE/CNFs and UHPLC-ESI-MS/MS.

	EIMTC Concentration [mol L^−1^] ± SD (n = 3)			Recovery [%]	Relative Error [%]	*t_exp._*
	Added	Found SWV	Found UHPLC-ESI-MS/MS			
Human	5.0 × 10^−8^	4.86 × 10^−8^ ± 0.17 × 10^−8^	5.21 × 10^−8^ ± 0.05 × 10^−8^	97.2	7.2	1.98
serum	1.0 × 10^−7^	0.99 × 10^−7^ ± 0.03 × 10^−7^	0.96 × 10^−7^ ± 0.03 × 10^−7^	99.0	3.0	0.71
